# Fungal spore involvement in the resuspension of radiocaesium in summer

**DOI:** 10.1038/s41598-018-37698-x

**Published:** 2019-02-13

**Authors:** Yasuhito Igarashi, Kazuyuki Kita, Teruya Maki, Takeshi Kinase, Naho Hayashi, Kentaro Hosaka, Kouji Adachi, Mizuo Kajino, Masahide Ishizuka, Tsuyoshi Thomas Sekiyama, Yuji Zaizen, Chisato Takenaka, Kazuhiko Ninomiya, Hiroshi Okochi, Atsuyuki Sorimachi

**Affiliations:** 1 Meteorological Research Institute, 1-1 Nagamine, Tsukuba, Ibaraki, 305-0052 Japan; 2 Graduate School of Science and Engineering, Ibaraki University, 2-1-1 Bunkyo, Mito, Ibaraki, 310-8512 Japan; 3 Center for Research in Isotopes and Environmental Dynamics, University of Tsukuba, 1-1-1 Tennodai, Tsukuba, Ibaraki, 305-8577 Japan; 4 Institute of Science and Engineering, Kanazawa University, Kakumamachi, Kanazawa, Ishikawa, 920-1192 Japan; 5 Department of Botany, National Museum of Nature and Science, 4-1-1 Amakubo, Tsukuba, Ibaraki, 305-0005 Japan; 6 Faculty of Engineering and Design, Kagawa University, 2217-20 Hayashi-cho, Takamatsu, Kagawa, 761-0396 Japan; 7 Graduate School of Bioagricultural Sciences, Nagoya University, Furo-cho, Nagoya, 464-8601 Japan; 8 Graduate School of Science, Osaka University, 1-1, Machikaneyama, Toyonaka, Osaka, 560-0043 Japan; 9 Research Institute for Science and Engineering, Waseda University, 3-4-1 Okubo, Shinjuku, Tokyo, 169-8555 Japan; 10 Fukushima Medical University, 1 Hikariga-oka, Fukushima, 960-1295 Japan; 11 Present Address: Center for Research in Isotopes and Environmental Dynamics, University of Tsukuba and Graduate School of Science and Engineering, Ibaraki University, and formerly at Meteorological Research Institute, Ibaraki, Japan; 12 Present Address: Meteorological Research Institute and formerly at College of Science, Ibaraki University, Ibaraki, Japan

## Abstract

We observed the atmospheric resuspension of radiocaesium, derived from the Fukushima Dai-ichi Nuclear Power Plant accident, at Namie, a heavily contaminated area of Fukushima, since 2012. During the survey periods from 2012 to 2015, the activity concentrations of radiocaesium in air ranged from approximately 10^−5^ to 10^−2^ Bq per m^3^ and were higher in the warm season than in the cold season. Electron microscopy showed that the particles collected on filters in summer were predominantly of biological origin (bioaerosols), with which the observed radiocaesium activity concentration varied. We conducted an additional aerosol analysis based on fluorescent optical microscopic observation and high-throughput DNA sequencing technique to identify bioaerosols at Namie in 2015 summer. The concentrations of bioaerosols fluctuated the order of 10^6^ particles per m^3^, and the phyla Basidiomycota and Ascomycota (true Fungi) accounted for approximately two-thirds of the bioaerosols. Moreover, the fungal spore concentration in air was positively correlated with the radiocaesium concentration at Namie in summer 2016. The bioaerosol emissions from Japanese mixed forests in the temperate zone predominately included fungal cells, which are known to accumulate radiocaesium, and should be considered an important scientific issue that must be addressed.

## Introduction

Several years have passed since the March 2011 accident at the Fukushima Dai-ichi Nuclear Power Plant (FDNPP) operated by Tokyo Electric Power Company. Approximately 71% of Fukushima Prefecture is covered by forest (see Supplementary Fig. S1), and 44% of the forested area was contaminated with at least 10–30 kBq m^−2^ of ^137^Cs (corresponding to 1 mSv y^−1^ of excess exposure) by the accident^1^. The forest contamination by the FDNPP accident was most serious to the northwest^2,3^. This heavily contaminated (>0.5 MBq m^−2^ of ^137^Cs) forest area consists of 428 km^2^ (approximately 3% of the total area of Fukushima Prefecture; ca. 14,000 km^2^)^3^. Since the accident, the radiological contamination of the forested area by ^134^Cs and ^137^Cs (radiocaesium) has decreased mainly due to radioactive decay, and not by erosion or other environmental mechanisms^2^. Therefore, the forest ecosystem is a large radiocaesium reservoir^1,3^ and a potential secondary source of atmospheric radiocaesium^4^. The Chernobyl study^5^ listed three mechanisms of secondary radioactive aerosol emissions (resuspension); (1) wind-blown suspension, (2) suspension due to human activities involving the contaminated fugitive dust; and (3) forest fires. Although resuspension sometimes refers only to (1), herein, we use the term in a more comprehensive sense. Notably, the Fukushima contamination exhibits bioecological resuspension from the contaminated forest, a new type of resuspension.

We measured radiocaesium resuspension^6^ in the atmosphere at Kawamata and Namie, Fukushima Prefecture, after the accident^7,8^. In this area, which is 30 to 35 km northwest of the FDNPP and surrounded by heavily contaminated forest, as defined above, the effects of the primary emission of radiocaesium from the FDNPP likely ceased in fall 2011^9,10^; then, from 2012–2015, the radiocaesium activity concentration in the air slowly decreased, although seasonal fluctuations were observed, with increases during the warm season and decreases during the cold season (Supplementary Fig. S2). At Namie, the average summer concentration (June–August 2013–2014) was approximately 6 times the average winter concentration (December–February 2013–2014). This seasonal pattern is the opposite that observed in urban areas^8–11^, but emission inventory calculations with an aerosol transport model have shown that direct/delayed primary emissions from the FDNPP cannot explain the seasonal fluctuations in 2013^4^. Monthly radiocaesium activity concentrations (September 2012 to December 2014) at a site in Namie close to that used in this study were previously reported^12^. The study showed summer maxima for both the ^137^Cs concentration and the coarse particulate fraction (>1.1 μm) that support our radiocaesium record. However, the study attributed the seasonal trends to changes in the prevailing local wind direction and the distribution of surface contamination.

Optical microscopic observations suggested that the radiocaesium host particles in summer were fugitive dust (numerous coarse particles); their presence was initially attributed to the fact that no aerosol size cutoff was applied during high-volume (HV) aerosol sampling, but the radiocaesium host particles were subsequently shown to be of biological origin^8^ (also see Supplementary Fig. S3). Biological origin particles, which include microorganisms, pollen, animal debris, and plant debris, are defined as bioaerosols. The bioaerosol concentrations and the ^137^Cs activity concentration determined by the filter samples from Kawamata and Namie were both high in the warm season and low in the cold season, and these results suggested that bioaerosols may play an important role in radiocaesium resuspension during the warm season^8^. The previous work also suggested^8^ a correlation between the ^137^Cs concentration and air temperature from August to September. A 3D aerosol transport model with soil dust resuspension^7^ and forest ecosystem emission schemes was employed to analyse the source and budget of radiocaesium in the air and showed that the resuspension of contaminated dust from the bare soil could not explain the summertime atmospheric radiocaesium level^4^.

In this study, we examined the bioecological resuspension of radiocaesium and the composition of the bioaerosols that serve as host particles at Namie in August and September 2015. Fungi are known to accumulate radiocaesium, which they incorporate as analogue of potassium^13,14^, and a very high radiocaesium concentration (629 Bq g^−1^ dry weight) was reported in fungal spores^15^. Therefore, we hypothesized that contaminated fungal spores may primarily account for the increased resuspension of radiocaesium during the summer. Here, we present novel data on the bioaerosols and the radiocaesium contamination of fungal spores and examine the associated relationships.

## Results

**Figure 1:**
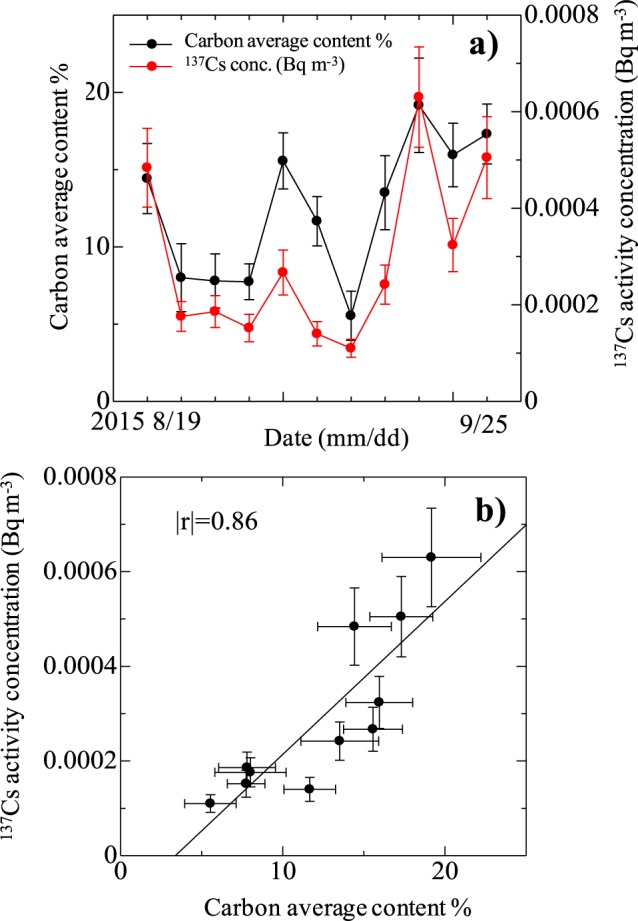
(**a**) Time series and (**b**) scatter plot of ^137^Cs activity concentrations and the average carbon content (area-averaged relative percentage) in August and September 2015. Carbon data were obtained by scanning electron microscopy coupled with energy-dispersive X-ray spectroscopy. Error bars indicate the measurement error (1σ). The good correlation between the two parameters suggests that organic particles (bioaerosols) are carriers of radiocaesium.

At Namie, the activity level of ^137^Cs in the air (based on HV aerosol sampling from 19 August to 25 September 2015) varied from approximately 100 to 600 μBq m^−3^, and this variation was coincident with that of the carbon content based on scanning electron microscopy coupled with energy-dispersive X-ray spectrometry (SEM-EDS) (Fig. 1). Due to the absence of heavy industrial and urban activities near the observation site, we inferred from this correlation that organic particles (bioaerosols) carry radiocaesium originating from the FDNPP accident.

**Figure 2:**
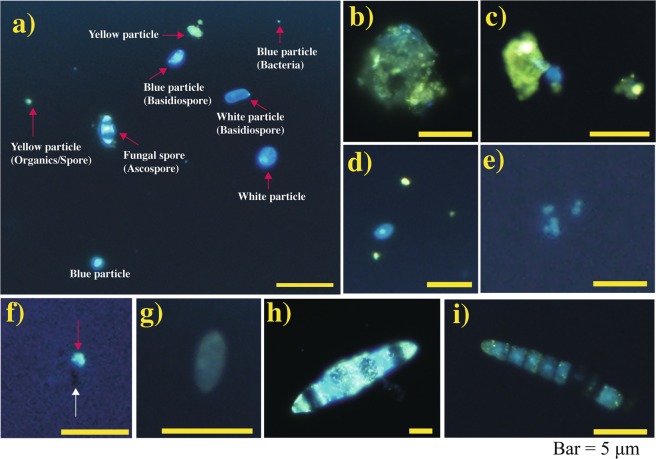
Fluorescent micrographs of DAPI-stained particles (indicated by red arrows) in the bioaerosol samples collected at Namie site in August and September 2015 (**a**). The aggregated particles observed as yellow particles (**b**,**c**), yellow and blue particles (**d**), blue particles (**e**), black (indicated by the white arrow) and white (indicated by the red arrow) particles (**f**), white particles (**g**) and spores form particles that are likely ascospores (**h**,**i**). The bars indicate a length of 10 μm. The assignment results were used to construct Fig. 3. In the photo, white and yellow particles may not exhibit the colours seen by the naked eye on the microscopic screen.

**Figure 3:**
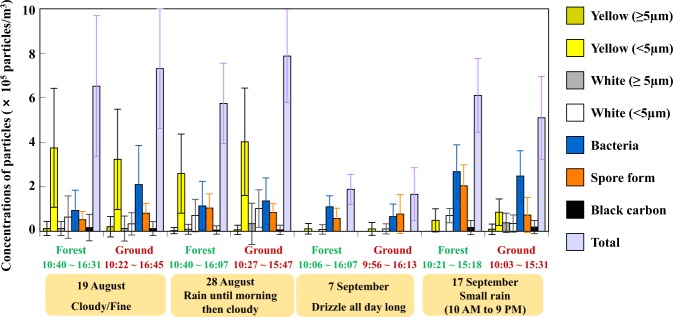
Number concentrations of DAPI-stained particles observed in the air samples collected from the forested and bare soil areas at Namie on 19 and 28 August and 7 and 17 September 2015, and the weather conditions on each sampling day. Particles have been classified by their colour and morphology: yellow particles ≥5 µm, organic aggregates; yellow particles <5 µm, organic particles/fungal spores; white particles ≥5 µm, mineral particles; white particles <5 µm, microbial particles; bacteria particles, bacteria; and black carbon particles, soot (so-called black carbon). Spore forms (orange bars), likely ascospores, were identified by morphology (see Fig. 2h,i).

**Figure 4:**
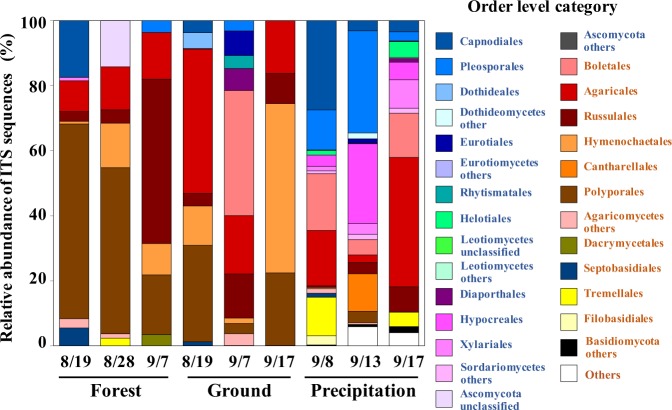
Results of the metagenomic analysis showing the relative abundance of identified fungal taxa in the samples collected at the forested and bare soil sites in Namie and contained in rain water collected at the site during summer 2015. Ascomycota are indicated in blue font, Basidiomycota in red font. Sampling dates are expressed as mm/dd. The compositional differences among sampling dates may reflect seasonal and weather differences.

We observed aerosol particles in bioaerosol samples collected during the sampling days using a fluorescent optical microscope observation with 4,6-diamidino-2-phenylindole (DAPI) staining. The fluorescent aerosol (FA) could be classified according to their fluorescence colour and morphology (Fig. 2). In general, the most abundant FAs were yellow particles (diameter <5 μm; indicating fungal cells/debris), blue particles (microbial particles), and particles identified as sporangia or ascospores. In particular, numerous particles with multiple septa, which are most likely fungal spores of the phylum Ascomycota, were observed. Only small amounts of white FA (<5 μm in size) and black aerosols, identified as mineral particles and black carbon, respectively, were observed. The total concentrations of FAs ranged from 1.7 × 10^5^ to 7.9 × 10^5^ particles m^−3^ (Fig. 3). Fewer yellow particles were observed in September than in August (Figs 3 and 4), possibly because of a seasonal change in the bioaerosol source or rainy weather on the sampling days in September (Supplementary Fig. S4 and Table S1). The total FA concentration differed little between forest and adjacent bare soil observation sites. The bioaerosol concentration ranged from 2 to 8 × 10^5^ particles m^−3^, of which 30 to 65% were of fungal origin.

High-throughput DNA sequencing analysis (Fig. 4 and Supplementary Tables S3 and S4) revealed that the sequences of the phylum Basidiomycota accounted for more than 80% in the total sequences of all aerosol samples, regardless of the land cover (forest or bare soil) at the observation site. In August, the members of the order Polyporales in Basidiomycota composed dominant communities in the forest, whereas Russulales sequences was dominantly detected in September. At the bare soil location, the members of the orders Agaricales, Boletales, Russulales, and Hymenochaetales in Basidiomycota were dominant in August. In September, rainwater samples exhibited larger proportions of Ascomycota, represented by the orders Capnodiales, Pleosporales, Dothidiales, Helotiales, Diaporthales, Hypocreales, and Xylariales, than did air samples. Ascomycota is the most species-rich phylum of Kingdom Fungi, and it includes numerous taxa with a prominent anamorphic (mould) stage during their life cycle^16^. Therefore, these results suggest that moulds were abundant in the observed environment.

**Figure 5:**
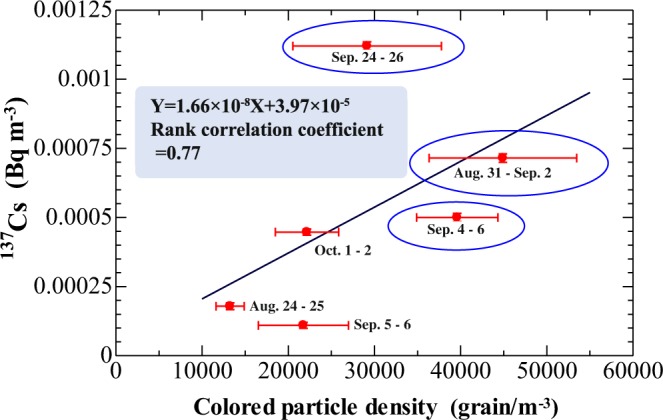
The relationship between the concentration of coloured fungal spores (countable without DAPI staining by optical microscopy) and the ^137^Cs activity in air at the Namie site in summer 2016. The sampling duration was 24 hours of daytime or nighttime (circled data; e.g., daytime data from Aug. 24–25 indicates that sampling was performed from 6:00 to 18:00 on August 24 and 25, a total of 24 hours). Despite the large scatter, the spore number and ^137^Cs concentration exhibited a positive correlation (rank correlation; significant at 8% based on a t-test). The slope of the fitted curve (1.66 × 10^−8^ Bq/grain) corresponds to the lower range of estimated values (see Supplementary Tables S5–S7).

We compared the number of coloured fungal spores (colourless spores were not counted) countable by optical microscopy (without DAPI staining) and the ^137^Cs activity in aerosol samples collected by an HV sampler at Namie in summer 2016 (Fig. 5). Sampling was conducted over 24 hours of daytime or nighttime (see the explanation of Fig. 5). Weather information on the 2016 sampling days is given in Supplementary Fig. S5 and Table S2. Although the data show considerable scatter, the correlation is relatively good in Fig. 5. The spore number concentration reached 5 × 10^4^ m^−3^, and the average ^137^Cs activity per fungal spore (grain), which is the slope of the correlation curve, was approximately 1.7 × 10^−8^ Bq/grain; this value is near the median of the estimated range (2.8 × 10^−9^ to 2.6 × 10^−7^) (Supplementary Information and Tables S5–S7). Some uncertainty (one order of difference) was associated with the spore number counting, as colourless spores were neglected (see the following discussion). Despite the uncertainty, the estimated and observed ^137^Cs activities in a single fungal spore were generally in good agreeance, which suggested that fungal spores are likely a significant atmospheric source of radiocaesium derived from the FDNPP accident, especially in late summer in the heavily contaminated forest area.

**Figure 6:**
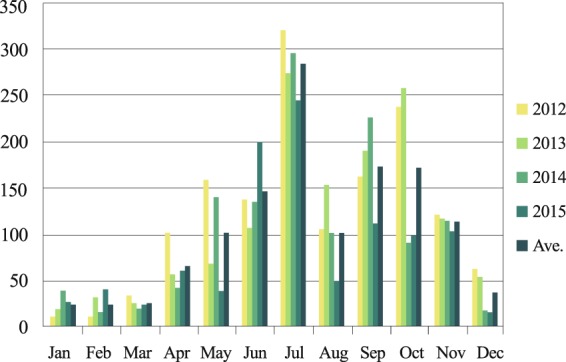
Monthly distribution of fungal specimens (both Basidiomycota and Ascomycota) collected at the Tsukuba Botanical Garden (Tsukuba, Japan) from 2012–2015 and the average values.

The monthly distribution of fungal specimens (fruiting bodies) collected from 2012–2015 at the Tsukuba Botanical Garden (36.10°N, 140.11°E, approximately 170 km southwest of FDNPP; area of ~140,000 m^2^; Fig. 6) supports our data on the fungal spore content of aerosols. The largest number of specimens was collected in July (all years), and the second largest number was collected in October (2012 and 2013) September (2014), and June (2015). In each year, the number of fruiting bodies collected was high from June–October, although fewer were collected in August. Additionally, relatively few fruiting bodies were collected from winter to early spring (December to March).

## Discussion

Recently, it was reported that in a temperate forest in Wakayama, Japan, approximately 3.5° latitude south of Namie in August 2010, fungal spores accounted for 45% of organic carbon aerosol at nighttime and 22% in the daytime, whereas biogenic volatile organic compound oxidation products accounted for 15% of organic carbon at nighttime and 19% during the daytime^17^. The results support our inference that in the forest at Namie, fungal spores rather than other bioaerosols are the major source of radiocaesium in the air in summer. The taxonomic composition varied even over the short observation period (Fig. 4), perhaps reflecting the seasonality of the fungal groups or the occurrence of rain. However, some members of Basidiomycota and Ascomycota would be the major carriers of radiocaesium at Namie.

A single sample of shiitake mushroom (*Lentinula edodes*) spores obtained in the northwestern evacuation area in 2014 was contaminated with 122 Bq g^−1^ dry weight of ^134^Cs and 629 Bq g^−1^ dry weight of ^137^Cs^15^. These concentrations are 1.9–9.0 and 2.5–10.9 times, respectively, higher than those in the fruiting bodies, suggesting radiocaesium bioaccumulation in fungal spores. It is probable that other fungi in the heavily contaminated area have similar radiocaesium activity levels in their spores.

These data and various other assumptions were used for the estimation that, on average, the ^137^Cs activity per fungal spore (Supplementary Tables S5–S7) ranges from 2.8 × 10^−9^ to 2.6 × 10^−7^ Bq/grain (see Methods and Supplementary Information). We should also note that the ^137^Cs concentration frequency distribution in fungi is very long tailed^14^. Using the ^137^Cs activity in shiitake mushroom spores (629 Bq g^−1^ dry)^15^, the weight of a single basidiospore (spore produced by Basidiomycota; 33 pg) and the weight of a single ascospore (spore produced by Ascomycota; 65 pg) reported in the literature^18^, we estimated ^137^Cs activity values of 2 × 10^−7^ and 4 × 10^−7^ Bq/spore, respectively. Considering the decrease in the ^137^Cs air concentration in each year (Supplementary Fig. S2), similarly, the ^137^Cs activity in a single spore would become lower annually. In our data, the slope of the relationship between the number of coloured fungal spores and the ^137^Cs concentration (Fig. 5), approximately 1.7 × 10^−8^ Bq/grain, is one order of magnitude lower than the abovementioned value, although it remains in the estimated range (Supplementary Tables S5–S7). The total fungal spore concentration, including both coloured and colourless spores, might be approximately one order of magnitude larger based on the data shown in Fig. 3. We have no reason to assume that coloured and colourless fungal spores have different mechanisms of emission, and they should move through the air in a similar manner and to similar extents. In this case, the ^137^Cs activity in a spore (the slope of Fig. 5) might be on the order of 10^−9^ Bq/grain as a mixture of coloured and colourless spores, which is also within the estimated range. These results strongly support fungal spore involvement in the resuspension of radiocaesium in the forested area at Namie during summer (bioecological resuspension).

Using a 3D aerosol transport model, the radiocaesium resuspension flux at Namie in summer 2013 was estimated^4^ to be approximately 22 mBq m^−2^ h^−1^. Spores with a radiocaesium content of 2.8 × 10^−9^ to 2.6 × 10^−7^ Bq/grain must be released from the forest at a rate of 2.2 × 10^1^ to 2.4 × 10^3^ grains m^−2^ s^−1^ to produce this ^137^Cs flux. These values are similar to or an order of magnitude larger than the maximum spore emission rate from the forest (387 grains m^−2^ s^−1^; Table 2 of ref.^19^). These findings suggest that fungal spores in Japan potentially have extensive environmental impacts, though internal radiation exposure via radiocaesium inhalation should be negligible (see the Appendix in the Supplementary Information).

The high-throughput DNA sequencing analysis showed that not only macroscopic fruiting bodies (i.e., mushrooms, mostly Basidiomycota) but also moulds (mostly Ascomycota), especially during precipitation periods, could provide major sources of bioaerosols (Fig. 4). Many species of Ascomycota are known to be plant pathogens or endophytes (fungi living inside plant tissues), and hyphae and spores on the tissue surfaces may concentrate radiocaesium and emit it into the air when the spores are launched. In the rain samples collected in September, Ascomycota accounted for as much as approximately 65% of the fungal groups, suggesting that the emission mechanism may be weather dependent (dry or wet).

It has been reported that the fungal spore count in air is high in summer and low in winter at several places around the world^20,21^. A review^22^ also noted seasonal differences in the atmospheric fungal aerosol concentration. These findings are consistent with our results from the Tsukuba Botanical Garden (Fig. 6) for a temperate forest in Japan. We did not calculate the biomass of mushroom fruiting bodies because only the number of specimens, each with a varying number of fruiting bodies, was recorded. Although the number of specimens can only indirectly indicate the mushroom biomass, these data are nonetheless consistent with the findings based on independent observations, such as the high-throughput DNA sequencing analysis targeting gDNA extracted directly from forest bioaerosol samples (Fig. 4) and the fluorescence microscopic observation of aerosol particles (Fig. 2). These seasonal cycles were demonstrated using the global model^23^.

Although no intensive fungal survey has been conducted in Namie area, and no intensive metagenomic analyses have been conducted in the Tsukuba Botanical Garden, both areas share similar climatic pattern and vegetation type (dominated by Quercus serrata and Q. acutissima of Fagaceae family). It is therefore mycologically unrealistic to assume that fungal flora between Namie and Tsukuba are dramatically different. Species composition between two areas may slightly differ, but we can empirically assume that family- and genus-level compositions, and seasonal patterns of fruiting, between Namie and Tsukuba are almost identical. Several pieces of direct and indirect evidence support this assumption. For example, all major orders of mushrooms detected by metagenomic analyses in Namie area (depicted in Fig. 4) have been reported from the Tsukuba Botanical Garden. In addition, all mushroom species, though sampling effort is limited, collected as the form of fruit bodies from Namie area during the 2017–2018 season (ca. 40 specimens) have been identified as genera and/or species that are also present in the Tsukuba Botanical Garden. Also, fluorescence microscopic observation indicated the airborne fungal spores and bacterial cells of Namie are similar to those in Tsukuba Botanical Garden site (Supplementary Fig. S6). Supplementary Fig. S7 also demonstrate similarities of bioaerosols over Namie and Tsukuba during summer rainy period. Besides, literature reports match our findings and suggest that radiocaesium activity associated with the movement of fungal spores is high in summer and low in winter. In addition, the high humidity and rainy conditions of the Japanese summer may favour the emission of fungal spores into the air^24–28^.

In addition to fungal spores, one possible source of radiocaesium in the air is contaminated cedar pollen. At Namie, radiocaesium activity concentrations up to approximately 253 Bq g^−1^ dry weight were observed in cedar pollen from November 2011 to January 2012^29–31^, but by 2015, they had decreased to no more than 25.4 Bq g^−1^. Therefore, in recent years, cedar pollen has likely played a limited role in radiocaesium resuspension. Furthermore, in Japan, cedar pollen is emitted from late February to early May^32^; therefore, it would not have been a source of the radiocaesium at Namie in summer.

Considering other possible secondary bioecological sources of radiocaesium in the forest environment, radiocaesium contamination in pollen and bee honey was reported in Munich, Germany, following the Chernobyl accident (surface ^137^Cs contamination, 17.4 kBq m^−2^ in early May 1986)^33^. The highest ^137^Cs concentration in pollen (>1 Bq g^−1^) was recorded in May 1986, but this level rapidly decreased to approximately 0.2 Bq g^−1^ by July 1986. By considering the surface contamination level of 1.5 MBq m^−2^ at Namie^12^ and assuming that the pollen contamination would be proportional to the surface contamination level, a pollen contamination level of up to 20 Bq g^−1^ can be estimated. In northern Italy during the early 2000s, the ^137^Cs effective half life in honey was 1.25 years on average^34^. If the half life in pollen is similar to that in honey, then after 4 years, the concentration would be reduced to 1/10 of the original level. Therefore, the level of radiocaesium contamination in pollen in the heavily contaminated areas of Fukushima Prefecture would have been approximately 2 Bq g^−1^. In addition, we detected no appreciable pollen, such as during the counting of bioaerosol fluorescent particles, because the sampling season (August and September) did not coincide with the flower bloom season. A previous work^8^ counted relative numbers of bioaerosols in air (“pollen” and “bacteria” categories, the latter including “spores”) in the warm season using scanning electron microscopy (SEM), and the results indicated that the “pollen” concentration was 1/10 of the “bacteria” concentration or less (Figure 12 in ref.^8^). Nevertheless, the pollen contribution to radiocaesium resuspension should still be considered because of the large size of pollen grains (≥30 μm^20^). Thus, even a small number of pollen grains might carry a detectable amount of radiocaesium.

Although no heavy radiocaesium contamination of pollen other than cedar has been reported in Japan, the suspension of pollen lasts until June, except for pollen from gramineous plants (Poaceae), ragweed, wormwood, and Japanese hop emitted from August to October based on an allergy study^35^. Furthermore, a significant amount of pollen was not found in the present DAPI-stained FA analysis or direct optical microscope observations (see Supplementary Information). Radiocaesium transfer in forest and aquatic ecosystems was examined in Fukushima Prefecture, and ^137^Cs accumulation was found to occur in the following order: litter > detritivores > fungi > predators > plants > herbivores^36^. This result suggests that any plants in the forest can accumulate radiocaesium as fungi. Previous work suggested that contaminated pollen grains may have contributed to an increase in the radiocaesium concentration in the air at Namie in May and June 2015^8^. During this early summer peak period, the radiocaesium concentration correlated with wind speed, which suggests a wind-blown source, such as pollen or fungal spores. In the future, year-round changes in the bioaerosol composition at Namie should be examined. In addition to mushrooms and moulds, lichens (mostly Ascomycota), algae, mosses, and bryophytes also produce microscopic spores, and lichens^37^ and mosses^38^ are known to amass radiocaesium. Other spore-producing organisms may also be candidate sources of bioecological radiocaesium resuspension. Furthermore, bacteria can accumulate radiocaesium^39,40^. Currently, we cannot exclude these other possible bioecological sources of radiocaesium resuspension.

Primary bioaerosols, including fungal spores, suspended in the atmospheric environment can have impacts on air quality^19,22,41^, agriculture^25^, and human health^42,43^. In addition, bioaerosols often act^44–48^ as ice-forming nuclei (IN) and cloud condensation nuclei (CCN). Thus, bioaerosols can have an appreciable effect on climate^22,41,48^. Previous reports of high fungal spore fluxes (1,000 or more spores m^−2^ s^−1^) have been limited to tropical and subtropical rainforest regions^22^, but the present findings suggest that even temperate-zone forests, such as those found in eastern Japan, can provide large sources of fungal spores and other bioaerosols. Our results are supported by those of a different study^49^, which demonstrated that the diversity of some groups of fungi (e.g., ectomycorrhizal mushrooms) in temperate and boreal areas equals or even exceeds those in tropical regions. The bioaerosols emission inventory in temperate forests should be investigated worldwide, as should the bioaerosol activity as IN and CCN in different regions. Furthermore, radiocaesium, as a useful chemical tracer, resuspension studies should also focus on the origins of other organic aerosols, such as humic-like substances and water-soluble organic compounds possibly sourced from primary bioaerosols.

## Methods

Atmospheric radiocaesium observations have been conducted in the contaminated area of Fukushima Prefecture since July 2011 (Supplementary Fig. S1). All sites are within 45 km to the northwest of the FDNPP and are inside the Planned Evacuation Area of 2011. Samples were collected using an HV aerosol sampler. The sampling locations and observations are described elsewhere in detail^4,7,8^. The activities of radiocaesium were measured at the Meteorological Research Institute (MRI) and at Osaka University by γ-ray spectrometry, following a procedure described elsewhere^8^. The morphology and elemental composition of aerosols collected on the filters were examined using SEM coupled with an energy-dispersive X-ray spectrometer (EDS), as well as a digital optical microscope (OM) with a data analyser.

Bioaerosols were sampled on sterilized polycarbonate filters at Namie from August—September 2015. Bioaerosols suspended in a few rain water samples were also collected on the filter by extracting a few tens of ml of the water by syringe. Bioaerosols on the filters were washed off with 1.5 mL of sterilized ultra-pure water containing 0.9% (w/v) of NaCl and shaken, and the solution samples were pelleted via centrifugation at 20,000 G. Genomic deoxyribonucleic acid (gDNA) was extracted using the combination of a phenol-chloroform extraction and the cell degradation by lysozyme, protease and sodium lauryl sulphate (SDS)^50^. Fragments of the internal transcribed spacer (ITS) region (approximately 400 base pairs; bps) were amplified from the extracted gDNA by polymerase chain reaction (PCR) using universal fungal primers ITS1-F -KYO1 (5′- Seq A - CTH GGT CAT TTA GAG GAA STA A -3′) and ITS2- KYO2 (5′- Seq B - TTY RCT RCG TTC TTC ATC -3′)^51^ for the ITS region. The first PCR fragments were amplified again using the second PCR primers, which targeted the additional sequences of the first PCR primers and included 8 tag nucleotides, such as Seq A and Seq B, designed for sample identification barcoding. Thermal cycling conditions were employed from a previous investigation^50^. PCR amplicons were used for high-throughput sequencing with a MiSeq Genome Sequencer (Illumina, CA, USA). The paired-end sequences with a read length of 461 bp were grouped based on the tag sequences of each sample. In the PCR analysis steps, negative controls (no template and template from unused filters) contained no fragments of ITS amplicons exhibiting the absence of contamination during the process. After the forward and reverse paired-end reads in the raw sequencing database were merged, the irregularly merged reads (lengths outside the 200–500 bp range or exceeding 6 photopolymers) and the error sequences with low Q-scores were removed. The remaining sequences were clustered into phylotypes using QIIME (Quantitative Insights Into Microbial Ecology; ver. 1.8.0) software with a minimum coverage of 99% and a minimum identity of 97%. The fungal compositions of the phylotypes were analysed using the Basic Local Alignment Search Tool (BLAST) to compare their sequences with references from the DNA Data Bank of Japan. Supplementary Tables S3 and S4 give numbers of ITS sequences classified into phylum and order, respectively. All sequences have been deposited in the DDBJ database (accession number of the submission is DRA007277).

We estimated the radiocaesium activity of a single fungal spore at Namie by assuming that the radiocaesium activity in fungi is proportional to the level of surface contamination. Potassium-40 concentration in fungi is often measured with the ^137^Cs activity, K content in fungi and ^40^K activity in the unit mass of K are known, and this approach could be employed to estimate the ^137^Cs content in a single fungal spore (Supplementary Tables S5–S7). In the calculation, we applied no decay correction for ^137^Cs due to its small effect on the estimation results. In the first and second approaches, fungal spores were assumed to be droplet and wooden particles, as shown in Supplementary Tables S5 and S6. The third approach (Supplementary Table S7) directly used the transfer factor in a forest. The estimates obtained by the three approaches overlap (approach 1, 8.1 × 10^−9^ to 7.8 × 10^−8^ Bq/grain; approach 2, 2.8 × 10^−9^ to 1.5 × 10^−7^ Bq/grain; and approach 3, 3.3 × 10^−9^ to 2.6 × 10^−7^ Bq/grain), which suggests that they are plausible and that the ^137^Cs content in a single fungal spore at Namie ranges from 10^−9^ to 10^−7^ Bq. The range of estimates mostly results from (1) the size (volume) difference of the basidiospore and ascospore fungal spores and (2) the difference in the ^137^Cs/^40^K activity ratios of fungi based on the level of surface contamination.

Monthly fungal fruiting body abundance levels were retrieved from a mushroom survey project at the Tsukuba Botanical Garden (Tsukuba, Ibaraki, Japan). The survey was conducted every week from 2012–2015. Fruiting bodies of both Basidiomycota and Ascomycota of visible size were surveyed and collected weekly from forested areas of the garden by 3 to 30 investigators. Here, a specimen is defined as one or more fruiting bodies of the same species growing in the same vegetation type (section) in the garden. On the same day, multiple specimens of the same species could be collected if they were found in different section of the garden. The total number of mushroom specimens collected each month, regardless of species, was counted, and the monthly average from 2012–2015 was calculated.

The Supplementary Information gives additional details of the above methods.

## Supplementary information

Supplementary Information
