# Supplementary material for: Fungal spore involvement in the resuspension of radiocaesium in summer
Source: Sci Rep. 2019 Feb 13;9:1954. doi: 10.1038/s41598-018-37698-x (PMC6374464; doi:10.1038/s41598-018-37698-x)
Supplement: Supplementary file 1 — Supplementary Information [file 41598_2018_37698_MOESM1_ESM.pdf]

Supplementary Information for
Fungal spore involvement in the resuspension of radiocaesium in summer

Yasuhito Igarashi[1,2,3,†,*], Kazuyuki Kita[2*], Teruya Maki[3], Takashi Kinase[1,2,‡], Naho Hayashi[2], Kentaro Hosaka[4], Kouji Adachi[1], Mizuo Kajino[1], Masahide Ishizuka[5], Tsuyoshi Thomas Sekiyama[1], Yuji Zaizen[1], Chisato Takenaka[6], Kazuhiko Ninomiya[7], Hiroshi Okochi[8], Atsuyuki Sorimachi[9]

[1] Meteorological Research Institute, 1-1 Nagamine, Tsukuba, Ibaraki 305-0052, Japan
[2] College of Science, Ibaraki University, 2-1-1 Bunkyo, Mito, Ibaraki 310-8512, Japan
[3] Center for Research in Isotopes and Environmental Dynamics, University of Tsukuba, 1-1-1 Tennodai, Tsukuba, Ibaraki 305-8577, Japan
[4] Graduate School of Natural Science and Technology, Kanazawa University, Kakuma, Kanazawa 920-1192, Japan
[5] Department of Botany, National Museum of Nature and Science, 4-1-1 Amakubo, Tsukuba, Ibaraki 305-0005, Japan
[6] Faculty of Engineering, Kagawa University, 2217-20 Hayashi-cho, Takamatsu, Kagawa 761-0396, Japan
[7] Graduate School of Bioagricultural Sciences, Nagoya University, Furo-cho, Nagoya 464-8601, Japan
[8] Graduate School of Science, Osaka University, 1-1, Machikaneyama, Toyonaka, Osaka 560-0043, Japan
[9] Research Institute for Science and Engineering, Waseda University, 3-4-1 Okubo, Shinjuku, Tokyo 169-8555, Japan
[10] Fukushima Medical University, 1 Hikariga-oka, Fukushima 960-1295, Japan
[†] Currently at Center for Research in Isotopes and Environmental Dynamics, University of Tsukuba and College of Science, Ibaraki University, and formerly at Meteorological Research Institute
[‡] Currently at Meteorological Research Institute and formerly at College of Science, Ibaraki University and Hitachi Power Solutions Co., Ltd.

* Corresponding author email: yasuhito.igarashi.sci50@vc.ibaraki.ac.jp (Y.I.) and kazuyuki.kita.iu@vc.ibaraki.ac.jp (K.K.)

This file contains:

Supplementary Methods

Supplementary Figures S1 to S7
Supplementary Tables S1 to S7
Appendix
Appendix Table 1
Supplementary and Appendix References

Supplementary Methods

Aerosol sampling. Atmospheric radiocaesium observations have been conducted at four locations in Fukushima Prefecture since July 2011: inside the forest and at a nearby point on the bare soil with/without decontamination in Yamakiya District, Kawamata town, and in Tsushima District, Namie town[1]. All four sites are no greater than 45 km to the northwest of the Fukushima Dai-ichi Nuclear Power Plant (FDNPP) and all are inside the Planned Evacuation Area of 2011. The sampling and the FDNPP locations are plotted on the land use map given in Supplementary Figure 1. The present observation sites are surrounded by deciduous and evergreen forests with irregular grasslands. The aerial ^{137}Cs average level of contamination was approximately 0.5 – 3 MBq m^{-2} in a 20 km range[2]. Radionuclide activity concentrations in aerosols were observed approximately 1.3 m above ground level every 4 to 14 days using a high-volume air sampler (Sibata HV 1000F and R, Tokyo, Japan and Kimoto model-120SL, Osaka, Japan). All sites exhibited almost the same seasonal trend in the radiocaesium air concentration. We focus on the Namie data in the present work. A pure SiO_2 fibre filter (Advantech QR100 or Pal flex 2500 QAT-UP; 203 mm × 254 mm, Tokyo, Japan) was used for bulk aerosol sampling (no aerosol size cut was applied). The sampling locations and observations are described elsewhere in detail[1,3,4].

In summer 2016, HV filter samples were collected separately during daytime and nighttime to determine the effect of meteorology on the resuspension of radiocaesium. However, discussing the effect of meteorology on the bioecological resuspension is beyond the scope of the present study and lacks sufficient data. Twenty-four-hour sampling was conducted over 2-3 days. For instance, daytime sampling on “Aug. 24-25” in Fig. 5 indicates collection occurred from 6:00 to 18:00 on August 24 and for the same duration on August 25. Additionally, the nighttime sampling on “Sep. 4-6” in Fig. 5 indicates that collection occurred from 18:00 on September 4 to 6:00 on September 5 and from 18:00 on September 5 to 6:00 on September 6.

Radioactivity measurement. The activities of ^{134}Cs and ^{137}Cs in the sampled aerosols were measured at the Meteorological Research Institute (MRI) and at Osaka University using intrinsic Ge detectors (coaxial-type from ORTEC EG&G, Eurisys or Canberra Japan, all from Tokyo, Japan) coupled with a computed multichannel spectrum analyser (Oxford-Tennelec Multiport, Niki Glass Company Ltd., Tokyo, Japan or Seiko EG&G MCA7600, Tokyo, Japan) following a procedure described elsewhere[1]. The detection limits for ^{134}Cs and ^{137}Cs at the MRI were approximately 9 and 10 mBq/sample, respectively, with a measurement period of 100,000 s, and at Osaka University, they were approximately 14 and 16 mBq/sample, respectively, with a measurement period of

250,000 s. Inter-laboratory comparison of the measurement data alleviated systematic data errors by using the same samples as calibrators (round robin samples). The measurement results are depicted in Supplementary Figure S2.

Aerosol morphology and composition. The morphology and elemental composition of aerosols collected on high-volume filters were examined using a scanning electron microscope (SEM) and a digital optical microscope (OM). The SEM used was SU3500 (Hitachi High-Technologies Co., Tokyo, Japan) coupled with an energy-dispersive X-ray spectrometer (X-max 50 mm^2, Horiba, Kyoto, Japan) under a low vacuum pressure (60 Pa) and with a maximum acceleration voltage of 20 kV. These conditions allowed the quartz fibre filters to be observed by SEM without any pretreatment, such as carbon coating. The OM employed was an Axio Imager M2m (Carl Zeiss, Tokyo, Japan) and used at 500 times magnification with a reflection mode. A portion of the filter samples was placed directly on a slide glass, which was observed with the OM. OM photos were taken with a CCD camera equipped with self-adjustment functions for white balance and exposure time. The obtained images were analysed with a photo retouching software. Supplementary Figure S3 gives OM and SEM images and the elemental maps provided by SEM-EDS of an HV filter sample obtained at Namie in summer.

Meteorological observations. Meteorological conditions may affect the species and concentrations of bioaerosols. Therefore, data were obtained by an automated weather station at the Namie bare ground site[3] on each sampling day and are summarized in Supplementary Table S1 and Figure S4. Similarly, meteorological data on sampling days in summer 2016 are given in Supplementary Table S2 and Figure S5.

Bioaerosol sampling. Bioaerosols were sampled at Namie on 19 and 28 August and 7 and 17 September 2015 based on the previously established method[5].

Bioaerosols were collected on sterilized polycarbonate filters (0.22 μm pore size; Whatman, Tokyo, Japan) with a sterilized filter holder connected to an air pump at a rate of ca. 0.0012 $m^3\ min^{-1}$. For each sample, two filters were used continuously during sampling periods ranging from 320 to 383 min (sampling air volumes ranged from 0.37 to 0.51 m^3). These filters were changed after each sampling period. Thus, eight air samples were obtained on the four sampling days. One of the two filters was used to determine particulate abundances by fluorescence microscopy, and the other was stored at –80°C until the extraction of genomic DNA (gDNA) to analyse the fungal community composition. Rainwater samples were also collected on 8, 13 and 17 September 2015. Bioaerosols suspended in the water were also collected on the filter by extracting a few tens of mL of the rain water using a syringe. The obtained residue on the filters was subjected to genomic analysis based the same method applied to the aerosol samples.

Fluorescent microscopic analysis of particle abundance. For fixing the microorganisms in bioaerosol samples, 0.25 mL of sterilized ultrapure water with paraformaldehyde at a final concentration of 1% was dripped on the filter with filter holder[6]. After 1 h of incubation in the paraformaldehyde solution, the aerosol particles were stained with 4,6-diamidino-2-phenylindole (DAPI) at a final concentration of 0.5 μg mL^{-1} for 15 min[7] and observed under an epifluorescence microscope (Olympus, Tokyo, Japan) equipped with an ultraviolet excitation system with 500-times magnification. A filter transect was scanned, and mineral particles (assigned white particles in Figs. 2 and 3; the same shall apply hereinafter), fungal particles (yellow particles), bacterial cells (blue particles), and fungal spores (spore form particles: sporangia/asci) along the filter transect were counted[8]. The detection limit of aerosols was below 1×10^4 particles m^{-3} of air. Fungal spores, especially those in the form of sporangia or ascospores, were interpreted as Ascomycota following the literature[9].

Pollen-like particles can rarely be observed by fluorescence microscopy because the sampling season (summer: August and September) does not coincide with the flower bloom period in forest areas. A previous publication[1] showed that bioaerosols are more abundant in air in summer (July and August) than in winter (December and January) and that the relative concentration of "bacteria" and "pollen" can be determined separately. It should be noted that "spores" were counted in the "bacteria" category in the study. The relative abundance of "pollen" was one-tenth or lower than that of "bacteria" (Fig. 12 in Ref. 1). Accordingly, the pollen concentrations would have been lower than the detection limit under microscopic observation (～10^4 per m^3) and were omitted from DAPI fluorescence counts.

Supplementary Figure S6 exhibits fluorescent aerosols collected at the Tsukuba Botanical Garden displaying the similarities of the bioaerosols between Namie and Tsukuba in summer season. Also, Supplementary Figure S7 demonstrate similarities of bioaerosol suspended during summer rainy period between Namie and Tsukuba. Currently intensive fungal metagenomic analyses of soil and atmospheric DNA are ongoing in the Tsukuba Botanical Garden to confirm what stated above, but it is still premature to include those raw date in this manuscript.

Determination of fungal community structures by MiSeq sequencing analysis. Aerosol particles on the filters were suspended in 600 μL of a sterile 0.6% NaCl solution and then pelleted by centrifugation at 20,000 *G* for 5 min. Genomic DNA was extracted from the pellets with sodium dodecyl sulphate, proteinase K, and lysozyme and then purified by a phenol-chloroform extraction procedure, as previously described[10]. In the analysis of fungal communities, fungal internal transcribed spacer (ITS) regions were

amplified by polymerase chain reaction (PCR) using the primers 4 (5′- Seq A – TCC GCT TAT TGA TAT GC -3′) and 7 (5′- Seq B - ACT CGC CGT TAC TGA GGC AAT -3′) in a Program Temperature Control System PC-700 thermocycler (ASTEC, Fukuoka, Japan) under the following conditions: 21 cycles of denaturation at 94°C for 1 min, annealing at 52°C for 2 min, and extension at 72°C for 2 min. Fragments of targeted sequences in the PCR products were amplified again using a forward primer (5′- Adaptor C - xxxxxxxx - Seq A -3′) and reverse primer (5′- Adaptor D - Seq B -3′), where xxxxxxxx indicates 8 nucleotide sequence tags designed for sample identification barcoding. Adaptors C and D were used for the sequencing with a Genome Sequencer MiSeq instrument (Illumina, CA, USA). Twelve cycles of denaturation at 94°C for 1 min, annealing at 59°C for 2 min, and extension at 72°C for 2 min were performed. A MonoFas DNA purification kit (GL Sciences, Tokyo, Japan) was employed for PCR amplicon purification. Aliquots of PCR amplicons from individual samples were pooled in a single tube for MiSeq sequencing. The sequences obtained for each sample were grouped according to sequence tags. After removal of the tags, the average read length was 461 base pairs (bps). Negative controls (no template and a blank template) were prepared at all steps of the procedure beginning with the DNA extraction to check for any contamination. After the culture cells of the fungi *Bdjerkandera adasta* and the spore-forming bacteria *Bacillus subtilis* were used for DNA extraction, the gDNA amounts were found to be linearly related to the spore numbers. This result suggested that the DNA extraction system can be used to obtain the genomic DNA from microbial cells.

Before analysing the community structures, we removed sequences less than 200 bps in length, those with a paired-equivalent quality score (Q-score) of <25, those containing ambiguous characteristics, those with an uncorrectable barcode, and those without the primer sequence. After the removal of these sequences, actual numbers of ITS reads for one sample ranged from 21,253 to 592,038 reads. The remaining sequences were clustered into phylotypes using QIIME (Quantitative Insights Into Microbial Ecology; ver. 1.8.0) software with a minimum coverage of 99% and a minimum identity of 97%. Supplementary Tables 3 and 4 indicate numbers of ITS sequences classified into each phylum and order, respectively. The fungal compositions of the phylotypes were analysed using the Basic Local Alignment Search Tool (BLAST) for comparison with those from the DNA Data Bank of Japan. All sequences obtained in the present work have been deposited in the DDBJ database (accession number of the submission is DRA007277).

Estimation of the radiocaesium carried by a single fungal spore. We employed three approaches to estimate the radiocaesium carried by a single fungal spore (Supplementary Tables S5–S7) to evaluate the potential concentration range around the Namie site. We

used the ^{40}K data, which are often measured with ^{137}Cs, from fungi and the known content of K in fungi for this purpose. All estimations were based on an average to estimate the probable range. In the calculation, a ^{137}Cs decay correction was not applied because 5 years of decay would result in an approximately 10% error level for ^{137}Cs activity. Other uncertainties and factors involved are notably more influential than this source of error. Even after 7 years, the associated error level is 15%, which could still be disregarded for the purpose of this study. Assuming that radiocaesium in a fungal fruit body is proportional to the level of surface contamination and that radiocaesium in the spores is proportional to that in the fruit body and taking the ^{40}K and K contents in fungi and the ^{40}K activity in units of the mass of K, the ^{137}Cs in a single spore could be obtained by the following equations:

(^{137}Cs in a single spore; Bq/grain) = (Spore volume; m^{3}) × (K in fungi; g m^{-3}) × (^{40}K/K; Bq g^{-1}) × CF × (^{137}Cs /^{40}K in fungi)$_{FDNPP}$ (1)

and

(^{137}Cs /^{40}K in fungi)$_{FDNPP}$ = (^{137}Cs /^{40}K in fungi)$_{GFO}$ × (^{137}Cs aerial surface activity; Bq m^{-3})$_{FDNPP}$/(^{137}Cs aerial surface activity; Bq m^{-3})$_{GFO}$ (2)

where CF represents a ^{137}Cs bioconcentration factor from the fruit body to a spore or the ratio between the fruit body and spore and the subscripts FDNPP and GFO represent the values associated with the FDNPP accident and nuclear weapon global fallout, respectively.

In the first approach (Supplementary Table S5), we assumed a basidiospore to be a sphere (5 μm[9,11]) and an ascospore to be a cylinder with a diameter of 5 μm and a height of 10 μm, as observed by fluorescent/conventional OM (Fig. 2), filled with cellular fluid containing K of known concentration (22.3 g L^{-1} (0.57 M in asci[12])). Potassium contains 30.4 Bq g^{-1} of ^{40}K[13]. The average ^{137}Cs/^{40}K activity ratio in fungi in Japan due to global fallout is known[14], and we assumed that the fungal ^{137}Cs concentration is proportional to the areal ^{137}Cs surface activity (contamination; Bq m^{-2}). The ^{137}Cs inventory in Japanese forest soils was estimated[15] to be 1.7 ± 1.4 kBq m^{-2} in 2008. In the late 1980s and early 1990s, the estimate was approximately 2.6 kBq m^{-2} on average with decay correction. The surface contamination level around Namie after the FDNPP accident was estimated to range[2] between 0.5 and 3 MBq m^{-2}. Therefore, we multiplied the (^{137}Cs/^{40}K ratio in fungi)$_{GFO}$ by a factor of 700 or 1,000 to estimate the ^{137}Cs per unit mass in fungi at Namie. The CF was assumed to be unity (no bioconcentration occurs) based on the fact that one of the present authors (C.T.) obtained a ^{137}Cs concentration of a 4 g shiitake fruiting body sampled in Kawamata, Fukushima equal to 24 Bq g^{-1} dry weight and that of a 0.005 g spore equal to 25 Bq g^{-1} dry weight (contrary to the results

in the Ref.[16]). The first approach yielded a range of 8.1×10^{-9} to 7.8×10^{-8} Bq/grain. In addition, in the first case, a cellular fluid density of 1,000 kg m^{-3}, the single basidiospore and ascospore weights were 6.5 pg/grain and 20 pg/grain, respectively.

In the second approach (Supplementary Table S6), we assumed a spore to be a dried wooden particle. Additionally, the spore volume was assumed to equal that in the first calculation.

(^{137}Cs in a single spore; Bq/grain) = (Spore volume; m^3) × (Dried wood density; kg m^{-3}) × (K in dried fungi; g kg^{-1}) × ($^{40}K/K$; Bq g^{-1}) × CF × ($^{137}Cs/^{40}K$ in fungi)$_{FDNPP}$ (3)

Thus, the typical dry density of wood (1,500 kg m^{-3}), the K content in fungi (g kg^{-1} dry weight)[14], the $^{137}Cs/^{40}K$ activity ratio in fungi due to global fallout[14], the surface contamination ratio due to the FDNPP accident and preaccident global fallout (1,000), and the ^{40}K activity per unit weight of K (Bq g^{-1}) were multiplied to calculate the ^{137}Cs activity of a single fungal spore (Bq/grain). The CF was assumed to be unity, as shown in Table S5. The second approach yielded the range of 2.8×10^{-9} to 1.5×10^{-7} Bq/grain. In the second case, the single basidiospore and ascospore weights were 9.8 pg/grain and 30 pg/grain, respectively.

The third approach (Supplementary Table S7) used the aggregated transfer factor in a contaminated forest area in Fukushima Prefecture[17] (T_{ag}; radiocaesium concentration (Bq kg^{-1}) divided by the total areal activity of radiocaesium (Bq m^{-2}).

(^{137}Cs content in a single spore; Bq/grain) = CF × $T_{ag\text{-}FDNPP}$ × (^{137}Cs aerial surface activity; Bq m^{-3})$_{FDNPP}$ (3),

where $T_{ag\text{-}FDNPP}$ is an aggregated transfer factor for fungi after the FDNPP accident obtained in different areas of Fukushima Prefecture[18]. The obtained values ranged from 0.01 to 3.0 (geometric mean of 0.2) depending on the fungal taxon. The CF was assumed to equal 1, and the CF reported for shiitake[16] was also applied in the calculation. The third approach yielded the range of 3.3×10^{-9} to 2.6×10^{-7} Bq/grain.

The estimates obtained by the three approaches overlap, which suggests that they are plausible. It should be noted that the range of estimates mainly resulted from 1) the size (volume) difference of the fungal spores between basidiospores and ascospores and 2) the difference in the $^{137}Cs/^{40}K$ activity ratio of fungi depending on the level of surface contamination.

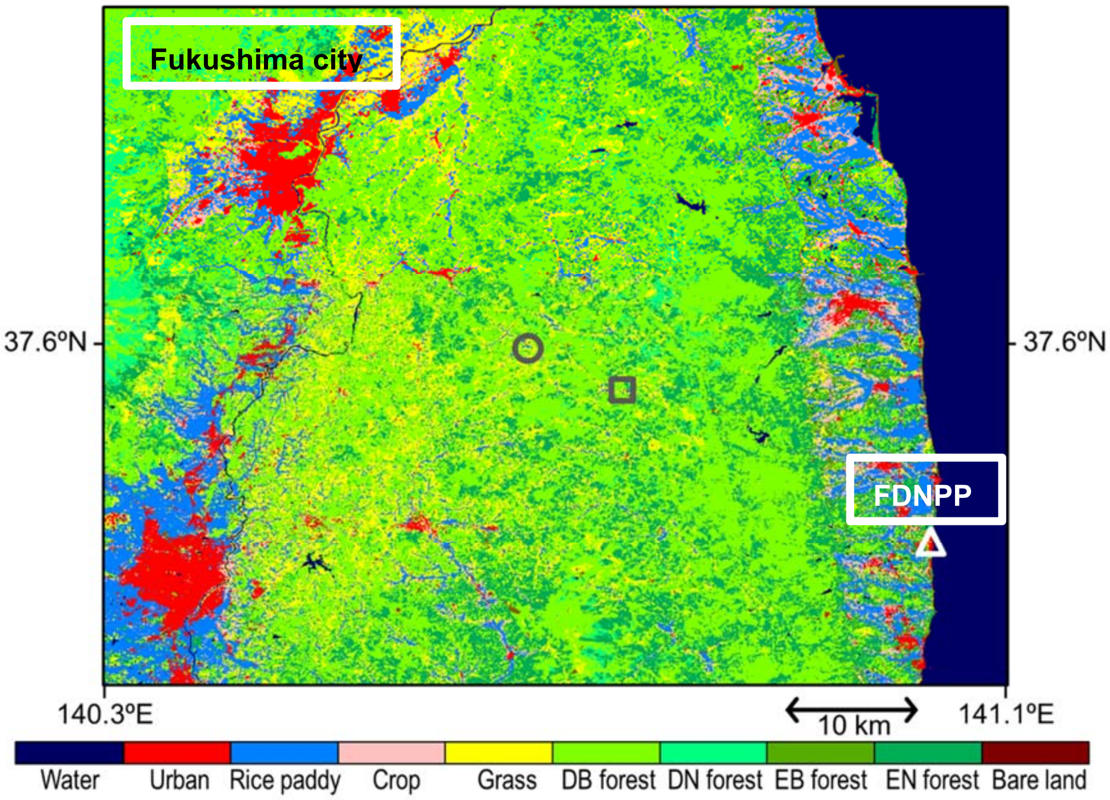

Supplementary Figure S1 Land cover map of eastern Fukushima Prefecture before the FDNPP accident. Triangle, FDNPP; circle, Kawamata site; square, Namie site; DB, deciduous broadleaved; DN, deciduous needleleaved; EB, evergreen broadleaved; and EN, evergreen needleleaved. The map has been modified from the literature[19]. It was created using data from the High-Resolution Land Use and Land Cover map published by the Japan Aerospace Exploration Agency Earth Observation Research Center ALOS/ALOS-2 Science Project and the Earth Observation Priority Research: Ecosystem Research Group, with permission granted.

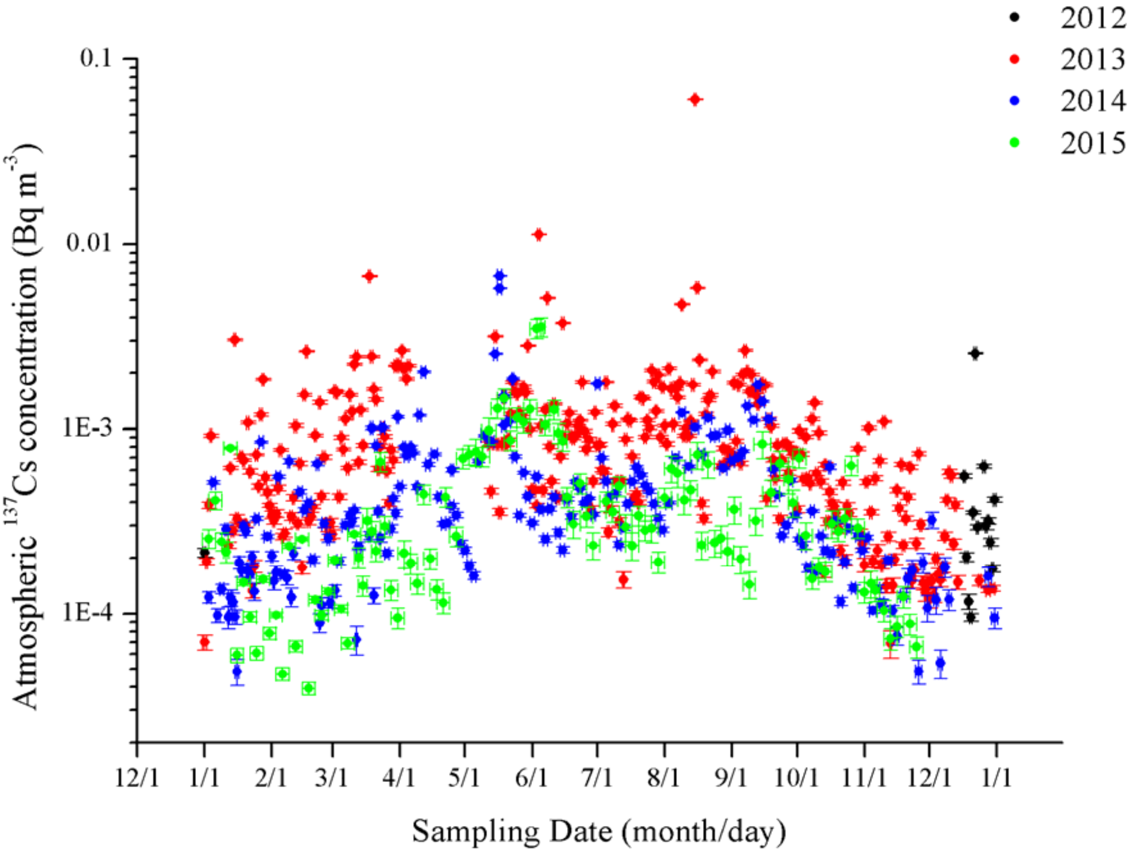

Supplementary Figure S2 Temporal changes in atmospheric ^{137}Cs concentrations observed at Namie in Fukushima Prefecture from 2012–2015 showing seasonal variations (the data from 2012–2014 were published in a previous work[1]). Horizontal and vertical bars for each value represent the sampling duration and measurement errors, respectively.

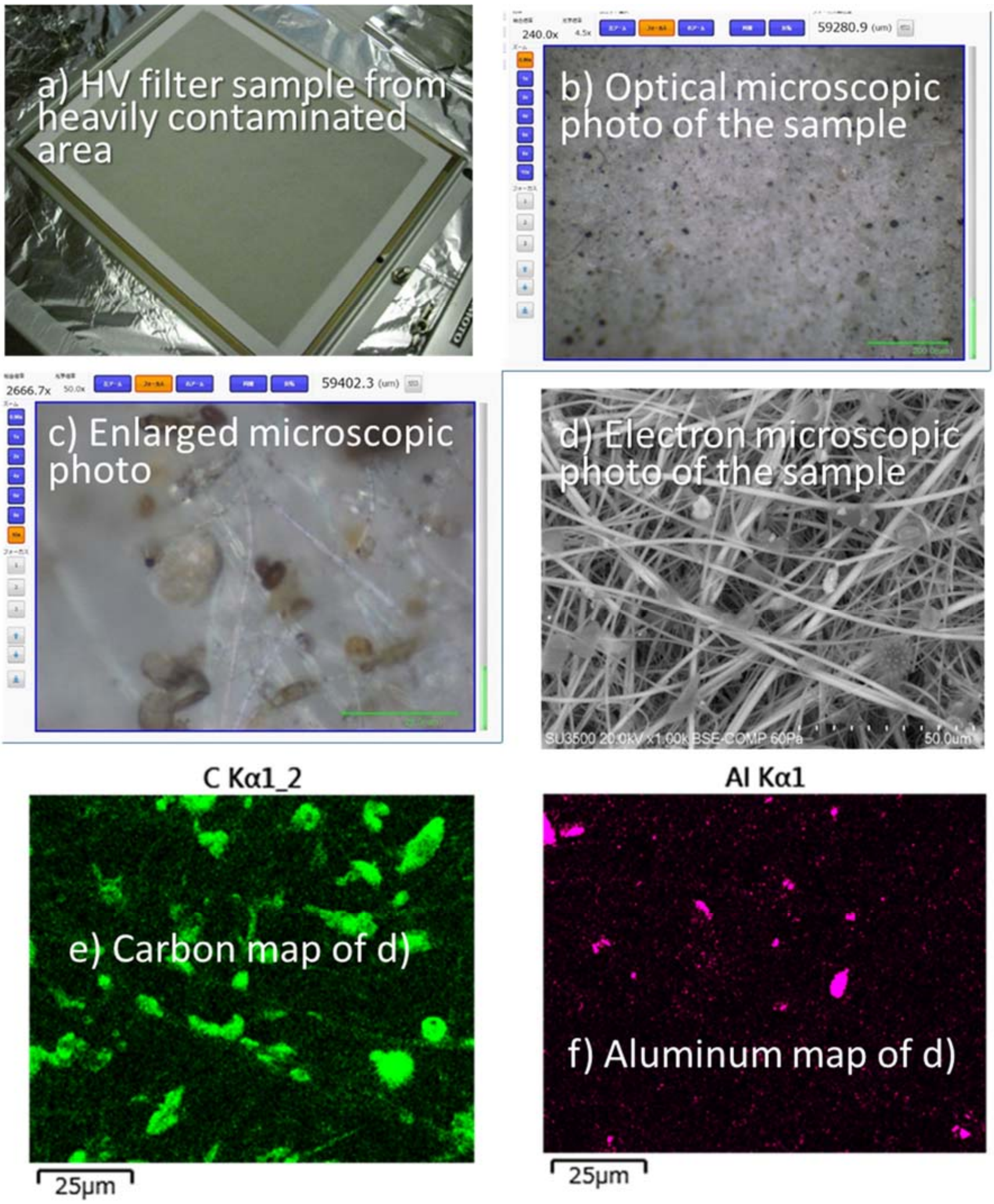

Supplementary Figure S3 Images of a high-volume quartz fibre filter used to collect aerosol samples during summer 2014 at Namie. a) Photograph of the filter showing the collected aerosols (grey- brown); b) optical microscopy photograph of the sample showing numerous coarse particles that appear to be fugitive dust c) enlarged optical microscopy image showing rounded particles and quartz fibres; d) SEM photograph; e) carbon map of d) showing abundant organic carbon/bioaerosol particles; and f) aluminium map of d) showing few mineral dust particles

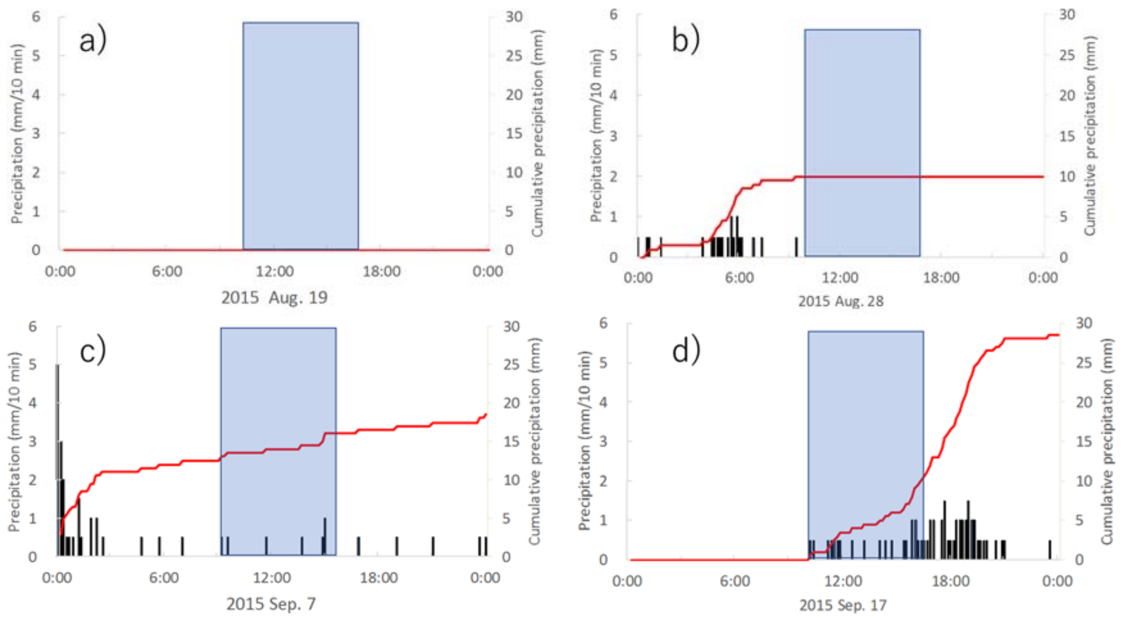

Supplementary Figure S4 Precipitation events (black bar) and cumulative precipitation (red line) at Namie on four sampling days in 2015: a) 19 August, b) 28 August, c) 7 September, and d) 17 September. The shaded bars show the sampling periods. In the cases of b, c and d), there was rain during the sampling period.

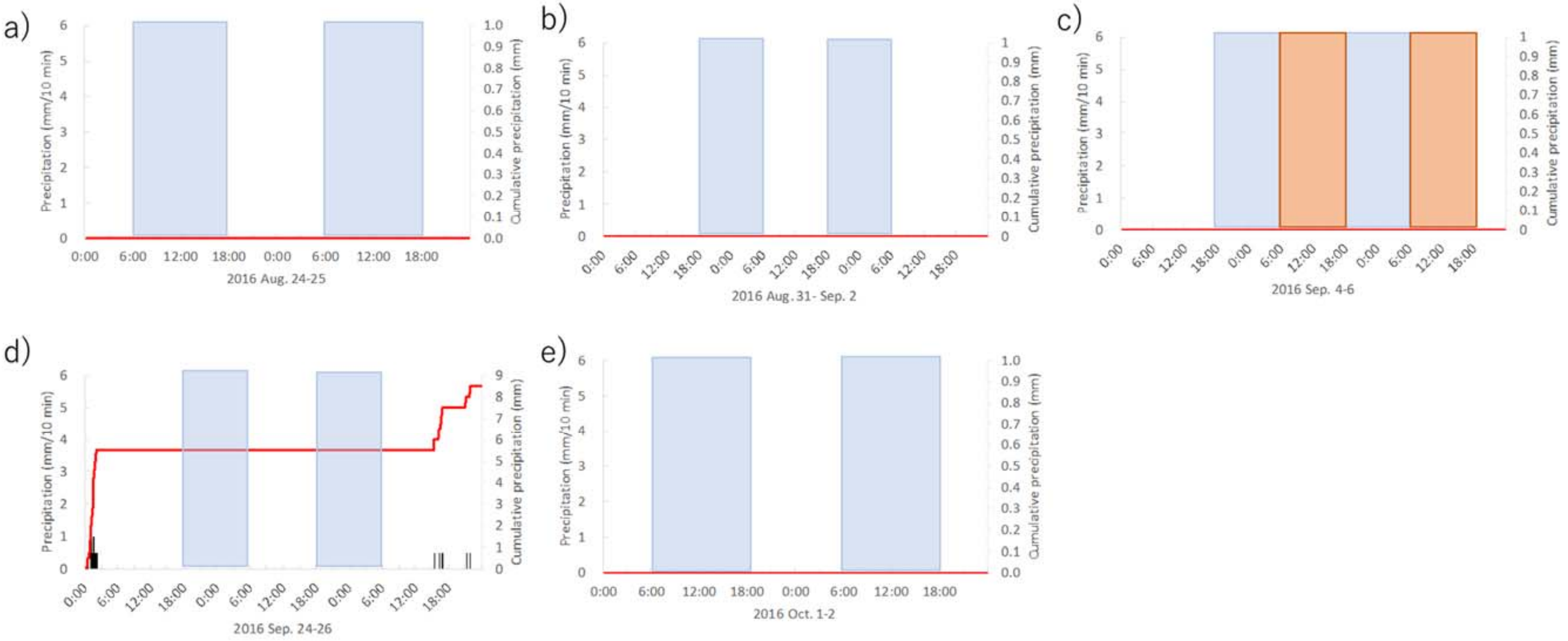

Supplementary Figure S5 Precipitation events (black bar) and cumulative precipitation (red line) at Namie on five sampling days in 2016: a) 24–25 August, b) 31 August–2 September, c) 4–5 September, and d) 24–26 September, and e) 1–2 October. The recording interval is 10 minutes. The shaded bars show the sampling periods. The sampling period c) included both daytime and nighttime (colour shad changes). In no case, evident rain events were observed.

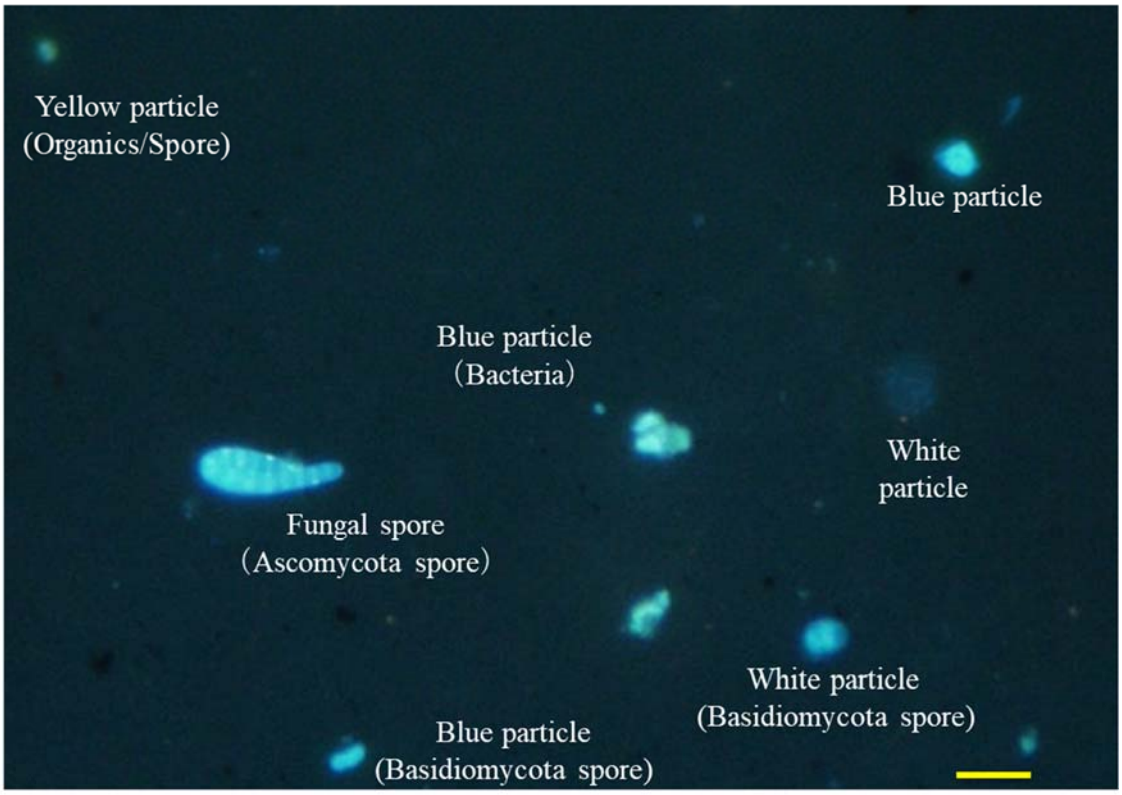

Supplementary Figure S6 Fluorescent micrographs of DAPI-stained particles in bioaerosol samples collected at the Tsukuba Botanical Garden site. Sampling was carried out during summer night (18 p.m. June 27 to 9 a.m. June 28, 2018). The sampling and fluorescent microscopic methods were identical to those employed for the Namie samples. Bars indicate the length of 10.0 µm. Similar fluorescent particles found at the Namie site, shown in Figure 2, are seen for this Tsukuba sample.

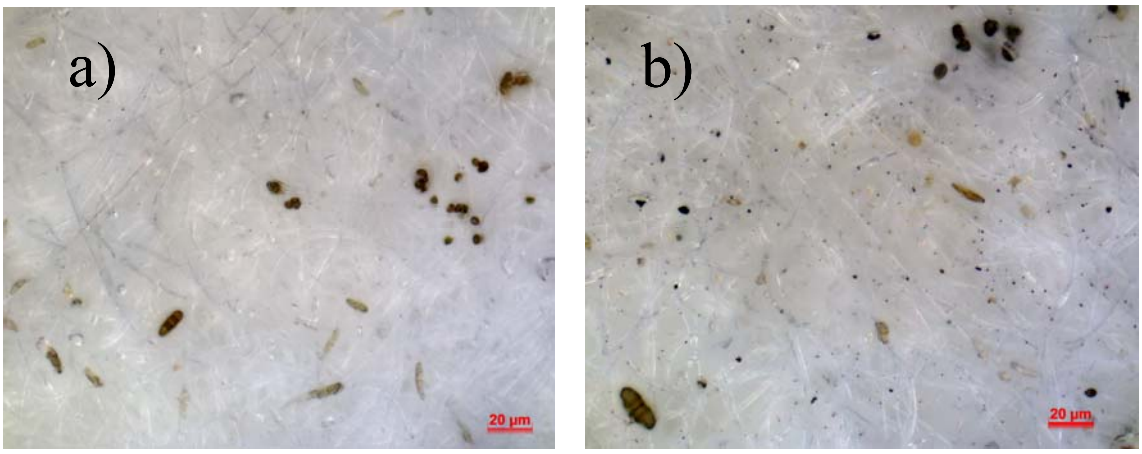

Supplementary Figure S7 Comparison of typical optical microscopic view of a) Namie and b) the Tsukuba Botanical Garden HV filter samples (with ×50 magnification in confocal mode) The former collected during rain period between June 14 to July 12, 2018 and the total air volume was 3391 m^3. While the latter collected during rain period between May 22 to June 5, 2018 and the total air volume was 850 m^3. With non-rain Tsukuba filter samples (collected during fine duration) too many particles pile up each other to identify the target bioaerosols, so those collected during rain are compared. Bioaerosols exhibiting similar morphology and colour are seen.

Supplementary Table S1 Mean values of meteorological parameters at Namie on bioaerosol observation days during summer 2015.

Items	Unit	Date (dd mmm.) in 2015			
		19 Aug.	28 Aug.	07 Sep.	17 Sep.
Wind speed	m s^{-1}	0.87	0.80	0.73	0.62
Air temperature	deg. C	25.8	18.1	17.7	15.9
Relative humidity	%	65	94	100	100
Soil temperature	deg. C	30.1	21.5	19.9	18.3
Soil moisture	m^3 m^{-3}	0.16	0.18	0.19	0.20
Precipitation	mm	0.0	0.0	2.5	7.0

Supplementary Table S2 Mean values of meteorological parameters at Namie on observation days during summer 2016.

Date<br>month. dd	Wind speed<br>$m\ s^{-1}$	Air temperature<br>deg. C	Relative humidity<br>%	Soil temperature<br>deg. C	Soil moisture<br>$m^3\ m^{-3}$	Precipitation<br>mm
Aug. 24	0.43	21.8	99.6	23.9	0.205	0.0
25	0.51	23.7	90.3	25.4	0.203	0.0
Aug. 31	0.49	21.1	88.0	23.2	0.207	0.0
1	0.34	21.1	89.4	23.2	0.200	0.0
2	0.44	20.7	93.6	23.0	0.198	0.0
Sep. 4	0.59	22.1	99.9	24.1	0.194	0.0
5	0.55	24.5	87.4	26.5	0.191	0.0
6	0.70	25.7	86.5	27.6	0.186	0.0
Sep. 24	0.26	18.1	98.9	19.7	0.213	5.5
25	0.41	19.8	95.3	21.0	0.211	0.0
26	0.12	19.7	99.7	20.4	0.213	3.0
Oct. 1	0.50	16.4	90.6	18.5	0.211	0.0
2	0.46	17.5	92.9	20.1	0.208	0.0

Supplementary Table S3 Numbers of ITS sequences for Namie samples which are classified into each phylum.

	Forest			Ground			Precipitation		
	19 Aug.	28 Aug.	7 Sep.	19 Aug.	7 Sep.	17 Sep.	8 Sep.	13 Sep.	17 Sep.
Ascomycota	109,614	72,426	13,745	9,670	67,318	3	43,434	14,315	26,777
Basidiomycota	482,413	437,404	363,345	100,720	245,497	147,426	48,818	5,694	63,388
Others	0	0	0	0	0	0	1	1,244	3,761

Supplementary Table S4 Numbers of ITS sequences for Namie samples which are classified into each order.

	Forest			Ground			Precipitation		
	19 Aug.	28 Aug.	7 Sep.	19 Aug.	7 Sep.	17 Sep.	8 Sep.	13 Sep.	17 Sep.
Capnodiales	103,208	46	0	4,107	0	0	25,321	671	3,239
Pleosporales	0	0	13,734	0	10,038	1	11,380	6,672	2,652
Dothideales	0	10	0	5,382	0	0	147	0	13
Dothideomycetes_others	112	143	0	4	0	0	1	389	153
Eurotiales	0	0	0	0	23,593	1	0	280	3
Eurotiomycetes_others	0	0	0	0	0	0	1	17	7
Rhytismatales	0	0	0	0	12,538	0	0	0	0
Helotiales	0	0	11	0	0	0	1,292	1	4,596
Incertae sedis	0	0	0	0	0	0	0	0	0
Leotiomycetes_others	0	0	0	1	0	0	0	0	277
Diaporthales	30	0	0	0	21,115	1	1	15	1,063
Hypocreales	0	31	0	4	0	0	3,236	5,212	5,113
Xylariales	6,240	0	0	0	0	0	1,214	710	8,195
Sordariomycetes_others	0	0	0	1	0	0	835	348	1,460
Incertae sedis	30	72,197	0	166	34	0	1	0	4
Ascomycota_others	0	0	0	4	3	0	6	0	3
Boletales	0	0	8	0	120,218	50	16,104	998	12,734
Agaricales	56,213	67,624	54,177	49,042	56,294	23,968	15,711	499	37,399
Russulales	17,548	20,827	190,668	4,238	42,321	13,642	482	732	7,263
Hymenochaetales	4,766	69,796	36,295	13,348	5,152	76,735	316	0	0
Cantharellales	0	0	0	1	0	0	1	2,456	41
Polyporales	354,695	260,365	68,985	32,744	9,866	32,930	1	746	19
Agaricomycetes_others	16,855	6,898	53	11	11,462	97	1,295	116	9
Dacrymycetales	0	0	13,164	0	181	4	0	0	0
Septobasidiales	32,342	5	0	1,324	0	0	1,130	0	0
Tremellales	0	11,894	0	13	0	0	10,941	1	4,143
Filobasidiales	0	0	0	0	0	0	2,593	0	2
Basidiomycota_others	0	0	0	0	0	0	246	146	1,776
Others	0	0	0	0	0	0	1	1,244	3,761

Supplementary Table S5 First estimation method of the ^{137}Cs concentration of a fungal spore in the heavily contaminated area for comparison with the actual measured values.

Volume of 5 μm⌀ sphere$^{\#}$ (m^3)	K conc. in cellular fluid ($g\ L^{-1}$)$^{\#3}$	$^{137}Cs/^{40}K$ in fungi* × the factor 700	^{40}K content in unit mass of K ($Bq\ g^{-1}$)	^{137}Cs activity per spore (Bq/grain)
6.54E-17	22.3	183	30.4	8.1E-09
		$^{137}Cs/^{40}K$ in fungi*2 × the factor 1000		
		262		1.2E-08
		$^{137}Cs/^{40}K$ in fungi*3		
		562		2.5E-08
Volume of 5 μm ⌀ bottomed cylinder with 10 μm length$^{\#2}$ (m^3)	K conc. in cellular fluid ($g\ L^{-1}$)$^{\#3}$	$^{137}Cs/^{40}K$ in fungi*2 × factor 1000	^{40}K content in unit mass of K ($Bq\ g^{-1}$)	^{137}Cs activity per spore (Bq/grain)
1.96E-16	22.3	262	30.4	3.5E-08
		$^{137}Cs/^{40}K$ in fungi*3		
		562		7.8E-08

Typical fungal spore diameter from the literature[9,11]

#2 Typical dimension of a fungal spore, presumably of Ascomycota, found in the present work (Figure 2)

#3 Potassium concentration found in ascus (0.57 M)[12]

* Caesium-137 and ^{40}K mean data in Japanese fungi[14] and the minimum factor of 700

*2 Caesium-137 and ^{40}K mean data in Japanese fungi[14] and the maximum factor of 1000

*3 Caesium-137 data[16] and ^{40}K data[14] are cited from different studies

Supplementary Table S6 Second estimation method of the ^{137}Cs concentration in a fungal spore in the heavily contaminated area for comparison with the actual measured values.

Volume of 5 μm⌀ sphere# (m^3)	Dry density (kg m^{-3})	K conc. in fungi (g kg^{-1} dry)$	^{137}Cs/^{40}K in fungi*× the factor 700	^{40}K content per unit mass of K (Bq g^{-1})	^{137}Cs activity per spore (Bq/grain)
6.54E-17	1.50E+03	3.00E+01	31.4	30.4	2.8E-9
			^{137}Cs/^{40}K in fungi*3		
			533		4.8E-08
Volume of 5 μm⌀ bottomed cylinder with 10 μm length#2 (m^3)		K conc. in fungi (g kg^{-1} dry) $	^{137}Cs/^{40}K in fungi*2 × factor 1000	^{40}K content per unit mass of K (Bq g^{-1})	^{137}Cs activity per spore (Bq/grain)
1.96E-16	1.50E+03	3.00E+01	31.4	30.4	8.4E-9
			^{137}Cs/^{40}K in fungi*3		
			562		1.5E-07

$ Potassium content was from the literature[14]

For others, the same conditions as those in Table 1 are applied.

Supplementary Table S7 Third estimation method for the ^{137}Cs concentration in a fungal spore in the heavily contaminated area based on the aggregated transfer factor (T_{ag}) approach. Basidiospores and ascospores are assumed to correspond to small yellow particles and large fungal spores, respectively (see Figure 2).

^{137}Cs deposition: 500 kBq m^{-2} *				^{137}Cs activity per spore (Bq/grain)	
RadioCs T_{ag} for mushroom fruit body (m^2 kg^{-1})	^{137}Cs conc. in fruit body (Bq g^{-1})	^{137}Cs conc. ratio of spore/fruit body (CF)	^{137}Cs conc. in fungal spores (Bq g^{-1})	Basidiospore (33 pg)	Ascospore (65 pg)
0.2$	100	1#	100	3.3E-09	6.5E-09
0.2	100	6.7%	670	2.2E-08	4.4E-08
^{137}Cs deposition: 3,000 kBq m^{-2} *2				^{137}Cs activity per spore (Bq/grain)	
RadioCs T_{ag} for mushroom fruit body (m^2 kg^{-1})	^{137}Cs conc. in fruit body (Bq g^{-1})	^{137}Cs conc. ratio of spore/fruit body (CF)	^{137}Cs conc. in fungal spores (Bq g^{-1})	Basidiospore (33 pg)	Ascospore (65 pg)
0.2	600	1#	600	2.0E-08	3.9E-08
0.2	600	6.7%	4020	1.3E-07	2.6E-07

* Lower range of surface contamination for ^{137}Cs near the Namie site[2]
*2 Higher range of surface contamination[2] for ^{137}Cs near the Namie site[2]
$ Geometric mean of radiocaesium T_{ag} for mushrooms and FDNPP radiocaesium[18]
# Data obtained by one of the present authors (C.T.) in Yamakiya
% The average CF value obtained from a shiitake mushroom (*Lentinula edodes*) sample[16]

Appendix

We estimated the effective dose for a person from the observed ^{137}Cs resuspension concentration at Namie for two assumed cases: a 1-day trip and a year-long stay. We assumed that bioaerosol particles carrying the ^{137}Cs were biologically recycled, moderately water soluble (based on an extraction experiment (unpublished data)), and slightly smaller in size than those observed (conservative assumptions). The effective dose A was then calculated as follows[20]:

$$A = C \times S \times Ka \times Q \times T,$$

where A is the effective dose (Sv);

C is the ^{137}Cs concentration in air (Bq m^{-3});

S is the time coefficient for the length of stay: (S1 + fc × S2) / 24 h) = 1/2, where S1 is the length of time spent outdoors each day (8 h), S2 is that spent indoors (16 h), and fc is the reduction coefficient (approximately 1/4);

Ka is the effective dose coefficient (2.90×10^{-8}, 1.80×10^{-8}, 1.30×10^{-8}, 1.10×10^{-8} of 9.70×10^{-9} Sv Bq^{-1} for a 1 year old, 5 year old, 10 year old, 15 year old, or adult (15–70 years old) member of the public[21];

Q is the air intake rate) (5.2, 8.7, 15, 20, or 22 m^{3} day^{-1} for a 1 year old, 5 year old , 10 year old, 15 year old, or adult[21]; and

T is the intake duration (1 day or 365 days).

As a typical value of C, we used the maximum ^{137}Cs concentration observed at Namie in 2014 (6750 μBq m^{-3}) and the maximum annual average ^{137}Cs concentration (546 μBq m^{-3}) from 2011–2014 (see Table 3 in the Supplementary Information and Appendix Ref. 1). Although the Namie ^{137}Cs data were generally free from the direct influence[4] of FDNPP primary emissions in a case that occurred in summer 2013[22], we took a conservative approach. The results (Table A1) show that staying for a single day or even an entire year should not result in exposure (effective dose) greater than the μSv level; therefore, the inhalation of resuspended ^{137}Cs would have a negligibly small health impact associated with the radiation.

Appendix Table A1 Estimates of inhalation exposure (effective dose) due to ^{137}Cs resuspension at Namie for a 1-day visit and a 1-year stay from 1-year-old children to adults

Age	^{137}Cs Concen. (Bq m^{-3})	Staying factor	Ka; Effective dose coefficients (Sv Bq^{-1})	Q; Air intake rate (m^3 day^{-1})	T: Dwell time (day)	A; Effective dose (μSv)
	Max. concentration					
1-year-old	6.75E-03	0.5	2.90E-08	5.2	1	5.09E-04
5-year-old	6.75E-03	0.5	1.80E-08	8.7	1	5.29E-04
10-year-old	6.75E-03	0.5	1.30E-08	15	1	6.58E-04
15-year-old	6.75E-03	0.5	1.10E-08	20	1	7.43E-04
Adult	6.75E-03	0.5	9.70E-09	22	1	7.20E-04
1-year-old	6.75E-03	0.5	2.90E-08	5.2	365	1.86E-01
5-year-old	6.75E-03	0.5	1.80E-08	8.7	365	1.93E-01
10-year-old	6.75E-03	0.5	1.30E-08	15	365	2.40E-01
15-year-old	6.75E-03	0.5	1.10E-08	20	365	2.71E-01
Adult	6.75E-03	0.5	9.70E-09	22	365	2.63E-01
	Max. annual average concentration					
1-year-old	5.46E-04	0.5	2.90E-08	5.2	365	1.50E-02
5-year-old	5.46E-04	0.5	1.80E-08	8.7	365	1.56E-02
10-year-old	5.46E-04	0.5	1.30E-08	15	365	1.94E-02
15-year-old	5.46E-04	0.5	1.10E-08	20	365	2.19E-02
Adult	5.46E-04	0.5	9.70E-09	22	365	2.13E-02

Supplementary and Appendix References

1 Kinase, T. *et al.* The seasonal variations of atmospheric $^{134,137}Cs$ activity and possible host particles for their resuspension in the contaminated areas of Tsushima and Yamakiya, Fukushima, Japan. *Progress in Earth and Planetary Science* **5**, 12 (2018).

2 Ochiai, S. *et al.* Temporal variation of post-accident atmospheric ^{137}Cs in an evacuated area of Fukushima Prefecture: Size-dependent behaviors of ^{137}Cs-bearing particles. *J. Environ. Radioact.* **165**, 131-139 (2016).

3 Ishizuka, M. *et al.* Use of a size-resolved 1-D resuspension scheme to evaluate resuspended radioactive material associated with mineral dust particles from the ground surface. *J. Environ. Radioact.* **166, Part 3**, 436-448 (2017).

4 Kajino, M. *et al.* Long-term assessment of airborne radiocesium after the Fukushima nuclear accident: re-suspension from bare soil and forest ecosystems. *Atmos. Chem. Phys.* **16**, 13149-13172 (2016).

5 Maki, T. *et al.* Variations in airborne bacterial communities at high altitudes over the Noto Peninsula (Japan) in response to Asian dust events. *Atmos. Chem. Phys.* **17**, 11877-11897 (2017).

6 Maki, T. *et al.* Variations in the structure of airborne bacterial communities in a downwind area during an Asian dust (Kosa) event. *Sci. Total Environ.* **488-489**, 75-84 (2014).

7 Porter, K. G. & Feig, Y. S. The use of DAPI for identifying and counting aquatic microflora1. *Limnol. Oceanogr.* **25**, 943-948 (1980).

8 Mostajir, B., Dolan, J. R. & Rassoulzadegan, F. A simple method for the quantification of a class of labile marine pico- and nano-sized detritus: DAPI Yellow Particles (DYP). *Aquat. Microb. Ecol.* **9**, 259-266 (1995).

9 Elbert, W., Taylor, P. E., Andreae, M. O. & Pöschl, U. Contribution of fungi to primary biogenic aerosols in the atmosphere: wet and dry discharged spores, carbohydrates, and inorganic ions. *Atmos. Chem. Phys.* **7**, 4569-4588 (2007).

10 Maki, T. *et al.* Phylogenetic diversity and vertical distribution of a halobacterial community in the atmosphere of an Asian dust (KOSA) source region, Dunhuang City. *Air Qual. Atmos. Health* **1**, 81-89 (2008).

11 Hoose, C., Kristjánsson, J. E. & Burrows, S. M. How important is biological ice nucleation in clouds on a global scale? *Environ. Res. Lett.* **5**, 024009 (2010).

12 Trail, F., Gaffoor, I. & Vogel, S. Ejection mechanics and trajectory of the ascospores of Gibberella zeae (anamorph Fuarium graminearum). *Fungal Genet. Biol.* **42**, 528-533 (2005).

13 International Atomic Energy Agency. *Natural activity concentrations and fluxes as indicators for the safety assessment of radioactive waste disposal* (International Atomic Energy Agency, 2005).

14 Yoshida, S. & Muramatsu, Y. Concentrations of radiocesium and potassium in Japanese mushrooms. *Environ. Sci.* **7**, 63-70 (1994).

15 Miura, S. *et al.* Towards prediction of redistribution of fallout radiocesium on forested area discharged from Fukushima Nuclear Power Plant. Geophysical Research Abstracts, EGU2015 8989 (2015).

16 Yamaguchi, T. *et al.* Autoradiography of the fruiting body and spore print of wood-cultivated shiitake mushroom (Lentinula edodes) from a restricted habitation area. *Mushroom Science and Biotechnology* **23**, 125-129 (2015).

17 Calmon, P., Thiry, Y., Zibold, G., Rantavaara, A. & Fesenko, S. Transfer parameter values in temperate forest ecosystems: a review. *J. Environ. Radioact.* **100**, 757-766 (2009).

18 Nakai, W., Okada, N., Ohashi, S. & Tanaka, A. Evaluation of ^{137}Cs accumulation by mushrooms and trees based on the aggregated transfer factor. *J. Radioanal. Nucl. Chem.* **303**, 2379-2389 (2015).

19 Ishihara, M. & Tadono, T. Land cover changes induced by the great east Japan earthquake in 2011. *Sci. Rep.* **7**, 45769 (2017).

20 NSC (former Nuclear Safety Committee). Kinkyu-ji Monitaringu Deta ni Motozuku Seryo Hyoka Houhou ni Tsuite (Teigen) dose evaluation method based on emergency monitoring data (recommendation). http://www.nsc.go.jp/anzen/shidai/genan2011/genan019/siryo2.pdf (in Japanese). (2011).

21 Eckerman, K., Harrison, J., Menzel, H.-G. & Clement, C. H. *Compendium of dose coefficients based on ICRP publication 60*, 2012).

22 Steinhauser, G. *et al.* Post-accident sporadic releases of airborne radionuclides from the Fukushima Daiichi Nuclear Power Plant site. *Environ. Sci. Technol.* **49**, 14028-14035 (2015).
